# Successful Cases of Combined Treatment for WIfI Clinical Stage 4 Chronic Limb-Threatening Ischemia Using Popliteal-to-Distal Bypass, Minor Amputation, Debridement, and Rheocarna Therapy in Hemodialysis Patients: A Case Series

**DOI:** 10.7759/cureus.106289

**Published:** 2026-04-01

**Authors:** Hiroto Yasumura, Kenichi Arata, Goichi Yotsumoto, Eiji Miyauchi, Atsuko Hiramine, Shousei Osako, Koji Ihara, Sawa Inoue, Shun Okamoto, Hideyuki Satozono, Koichiro Shimoishi, Yoshihiro Fukumoto, Yuki Ogata, Tomoyuki Matsuba, Yoshiharu Soga

**Affiliations:** 1 Cardiovascular Surgery, Kagoshima City Hospital, Kagoshima, JPN; 2 Cardiovascular Medicine, Kagoshima City Hospital, Kagoshima, JPN; 3 Plastic and Reconstructive Surgery, Kagoshima City Hospital, Kagoshima, JPN; 4 Cardiovascular Surgery, Graduate School of Medical and Dental Sciences, Kagoshima University, Kagoshima, JPN

**Keywords:** chronic limb-threatening ischemia, clti, distal bypass, rheocarna, wifi

## Abstract

Chronic limb-threatening ischemia (CLTI) is a severe form of lower extremity artery disease. CLTI is an umbrella term for conditions in which the lower limb is threatened by ischemia, tissue defects, neural disorders, or infection. In Japan, CLTI primarily affects patients with diabetes and those on hemodialysis. The severity of CLTI is assessed using the Wound, Ischemia, and foot Infection (WIfI) classification. CLTI needs multidisciplinary therapy. Rheocarna is a novel direct hemoperfusion device and can adsorb low-density lipoprotein (LDL) cholesterol and fibrinogen, thereby improving hemorheology. However, reports describing its use after distal bypass surgery are limited. In this report, we present three initial cases of successful combined treatment for WIfI clinical stage 4 CLTI in patients undergoing hemodialysis, including distal bypass, minor amputation, debridement, and Rheocarna therapy.

Case 1 is a 67-year-old man with CLTI in the left foot. Endovascular therapy (EVT) failed to revascularize the anterior tibial artery (ATA). Necrosis of the left fifth toe progressed, and the ulcerative purulent wound tested positive for *Pseudomonas aeruginosa* (W3I3fI1; stage 4). A left below-knee popliteal-to-dorsal artery in situ bypass was performed, and the necrotic fifth toe was amputated, followed by maintenance debridement. Rheocarna therapy was initiated on postoperative day (POD) 13 (a nonhemodialysis day following the fifth postoperative dialysis) and administered twice weekly for a total of 12 sessions. Three months after surgery, the wound healed. Case 2 is a 71-year-old man with CLTI and necrosis of the left fourth and fifth toes. EVT failed to revascularize the chronic total occlusion (CTO) at the distal ATA. The ulcerative purulent wound tested positive for *Escherichia coli* (W3I2fI3; stage 4). A left below-knee popliteal-to-posterior tibial artery (PTA) in situ bypass was performed, and the necrotic third and fifth toes were amputated. Maintenance debridement with negative pressure wound therapy was performed. Rheocarna therapy was initiated on POD 7 (a nonhemodialysis day following the third postoperative dialysis) and administered twice weekly for a total of eight sessions. Three months after surgery, the wound healed. Case 3 is a 79-year-old man with CLTI and necrosis of the right first and fifth toes. EVT failed to revascularize the CTO at the distal ATA and PTA. Necrosis worsened, complicated by cellulitis, and the purulent wound tested positive for *Staphylococcus aureus* (W2I3fI3; stage 4). A right below-knee popliteal-to-dorsal artery in situ bypass was performed, and the necrotic first and fifth toes were amputated, followed by maintenance debridement. Rheocarna therapy was initiated on POD 9 (a nonhemodialysis day following the fourth postoperative dialysis) and administered twice weekly for a total of nine sessions. Three months after surgery, the wound healed.

Rheocarna therapy may reduce postoperative fibrinogen, LDL cholesterol and lipoprotein(a), improving microcirculation and avoiding bypass graft failure, surgical site infection and delayed wound healing. Given the limited reports on the use of Rheocarna after distal bypass, it is important to accumulate more cases and long-term outcome data. Further multicenter studies or registry data are needed to validate the optimal timing and frequency of Rheocarna therapy.

## Introduction

Chronic limb-threatening ischemia (CLTI) is a severe form of lower extremity artery disease (LEAD). CLTI is an umbrella term for conditions where the lower limb is threatened by ischemia, tissue defects, neural disorders, and infection, all of which require immediate intervention [1]. In Japan, CLTI primarily affects patients with diabetes (70%-80%) and those on hemodialysis (50%) [2]. The severity of CLTI is assessed using the Wound, Ischemia, and foot Infection (WIfI) classification, which stages the affected foot [3].

WIfI clinical stage 4 is a critical stage, and one-year amputation rates are from 20% to 64% [4]. It is particularly challenging to treat, especially in patients with diabetes mellitus or those on hemodialysis. Distal bypass, minor amputation, and subsequent debridement are effective combined treatments aimed at avoiding major amputation. However, these patients are often prone to delayed wound healing, surgical site infection (SSI), and immediate bypass occlusion following surgery.

Rheocarna (Kaneka Corporation, Osaka, Japan) is a novel direct hemoperfusion device capable of adsorbing low-density lipoprotein (LDL) cholesterol (LDL apheresis) and fibrinogen, thereby improving hemorheology [5]. However, the extent to which Rheocarna can improve these parameters has not been well documented, and reports of its use following distal bypass surgery are rare [6]. Here, we report three initial cases of successful combined treatments for WIfI clinical stage 4 CLTI in patients undergoing hemodialysis, including popliteal-to-distal bypass, minor amputation of the toes, debridement, and Rheocarna therapy, along with a review of the literature.

## Case presentation

Patients and methods

**Figure 1:**
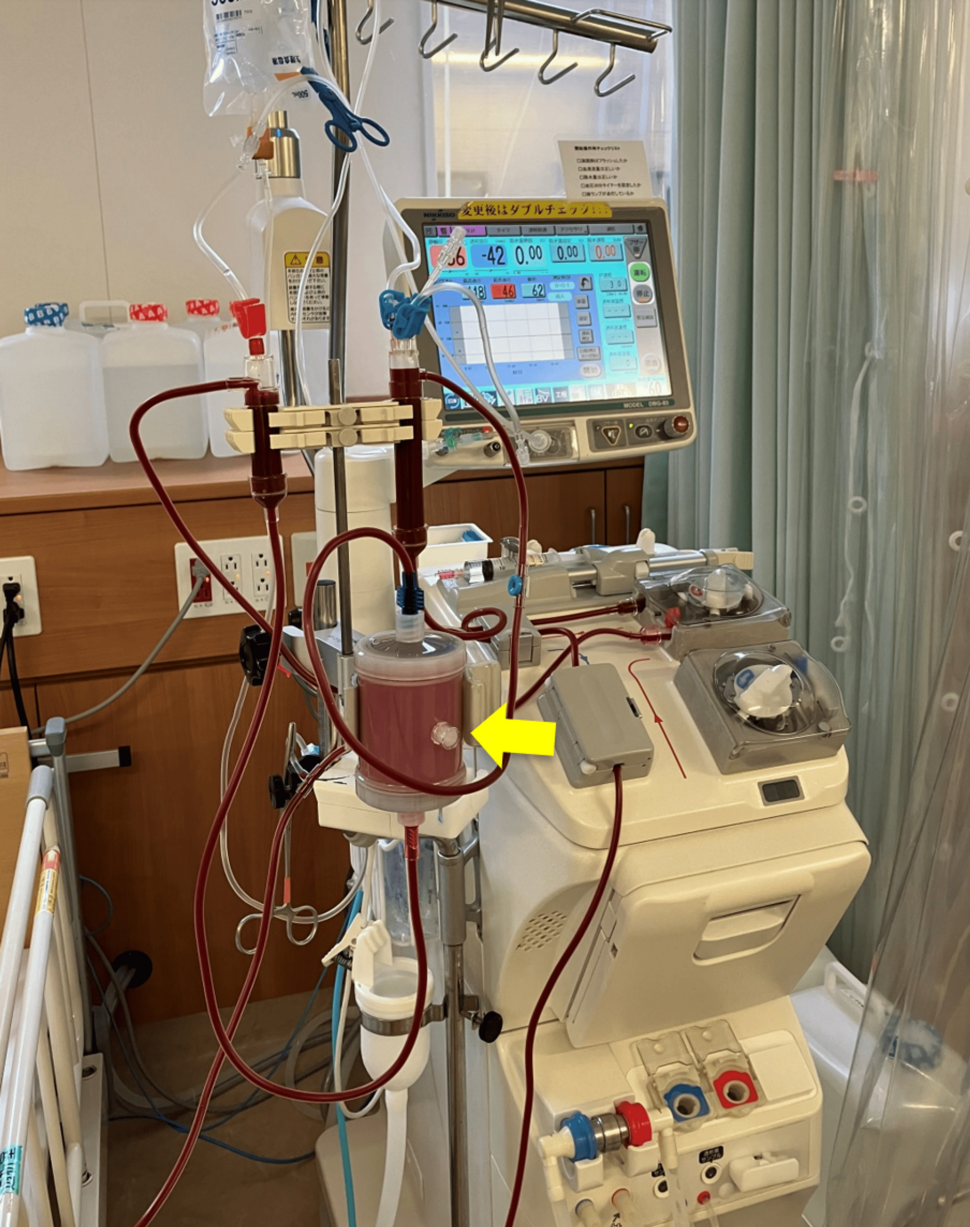
Rheocarna used in Cases 1-3 Rheocarna (yellow arrow) is a novel direct hemoperfusion device that adsorbs low-density lipoprotein cholesterol and fibrinogen, thereby improving hemorheology

This retrospective case series examined the clinical course and characteristics of three consecutive patients with WIfI clinical stage 4 CLTI who underwent combined treatment, including popliteal-to-distal bypass, minor toe amputation, debridement, and Rheocarna therapy (Figure 1) at Kagoshima City Hospital in Japan. These patients represented all cases treated after the introduction of Rheocarna in our department in 2024. Retrospectively collected data included patient demographics, physical examination findings, foot conditions (Table 1), presenting symptoms, and treatment course.

**Table 1:** Preoperative characteristics of three hemodialysis patients(Cases 1-3) with WIfI clinical stage4 CLTI, who received Rheocarna therapy BMI: body mass index; HD: hemodialysis; WBC: white blood cell; CRP: C-reactive protein; Alb: albumin; HbA1c: hemoglobin A1c; NGSP: National Glycohemoglobin Standardization Program; LDL-C: low-density lipoprotein cholesterol; ABI: ankle brachial index; SPP: skin perfusion pressure; DP: dorsal pedis; PP: plantar pedis; WIfI: Wound, Ischemia and foot Infection

Case	Age/sex	BMI (kg/m^2^)	Affected foot	HD period (years)	WBC (/μL)	CRP (mg/dL)	Alb (g/dL)	HbA1c (NGSP) (%)	Glycoalbumin (%)	LDL-C (mg/dL)	Fibrinogen (mg/dL)	ABI of the affected foot	SPP of the affected foot (mmHg)		WIfI classification
													DP	PP	
1	67/Male	23.1	Left	14	5,600	2.3	3.1	6.6	16.6	112	564	Unmeasurable	Not measured		W3I3fI1
2	71/Male	16.8	Left	4	13,000	8.9	3.3	7.9	30.3	84	Not measured	0.95			W3I2fI3
3	79/Male	17.7	Right	8	15,000	14	2.6	5.1	Not measured	74	527	0.83	Unmeasurable	14	W2I3fI3

Findings

Case 1

 A 67-year-old Japanese man (height: 162.3 cm; weight: 60.8 kg), who had been on hemodialysis for 14 years due to diabetic nephropathy, presented with intermittent claudication. He had a history of percutaneous coronary intervention. Contrast-enhanced CT revealed multiple vascular stenoses in the lower limbs, and he was referred to our hospital. Both ankle-brachial indexes were unmeasurable.

**Figure 2:**
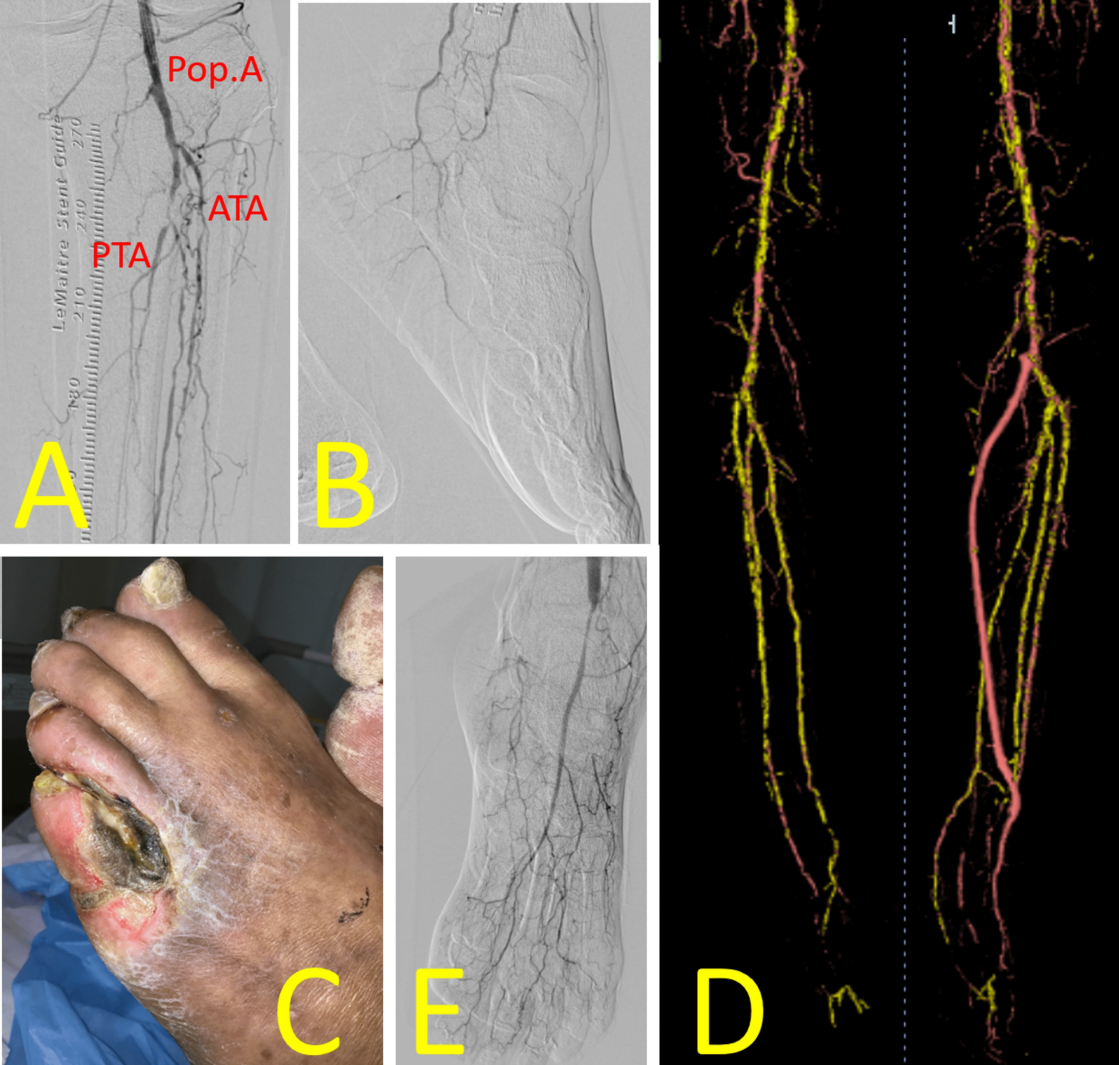
Perioperative findings of Case 1 (A) EVT to the left iliac and femoral arteries increased collateral blood flow to the lower limb. (B) The foot area supplied by the ATA remained avascular. (C) Despite EVT, necrosis of the left fifth toe progressed, with a red ring sign visible around the wound. (D) Left below-knee popliteal-to-dorsalis pedis artery in situ saphenous vein bypass was performed. (E) The bypass successfully increased blood flow in the ATA territory Image credit: These images were obtained at Kagoshima City Hospital as part of routine clinical care EVT: endovascular therapy; Pop. A: popliteal artery; ATA: anterior tibial artery; PTA: posterior tibial artery

Endovascular therapy (EVT) achieved revascularization of the left common iliac to common femoral artery from 90% to 25%, the mid-to-distal superficial femoral artery from 90% to 25%, and the popliteal artery from 90% to 25%, thereby increasing collateral blood flow to the left lower limb (Figure 2A). However, the foot area supplied by the anterior tibial artery (ATA) remained avascular (Figure 2B). Necrosis of the left fifth toe progressed (Figure 2C), and the purulent ulcerative wound tested positive for *Pseudomonas aeruginosa* (W3I3fI1; stage 4). Subsequently, we performed a left below-knee popliteal-to-dorsal artery in situ saphenous vein (SV) bypass (Figure 2D), resulting in successful revascularization (Figure 2E). The necrotic fifth toe was amputated, and weekly maintenance debridement was performed.

**Figure 3:**
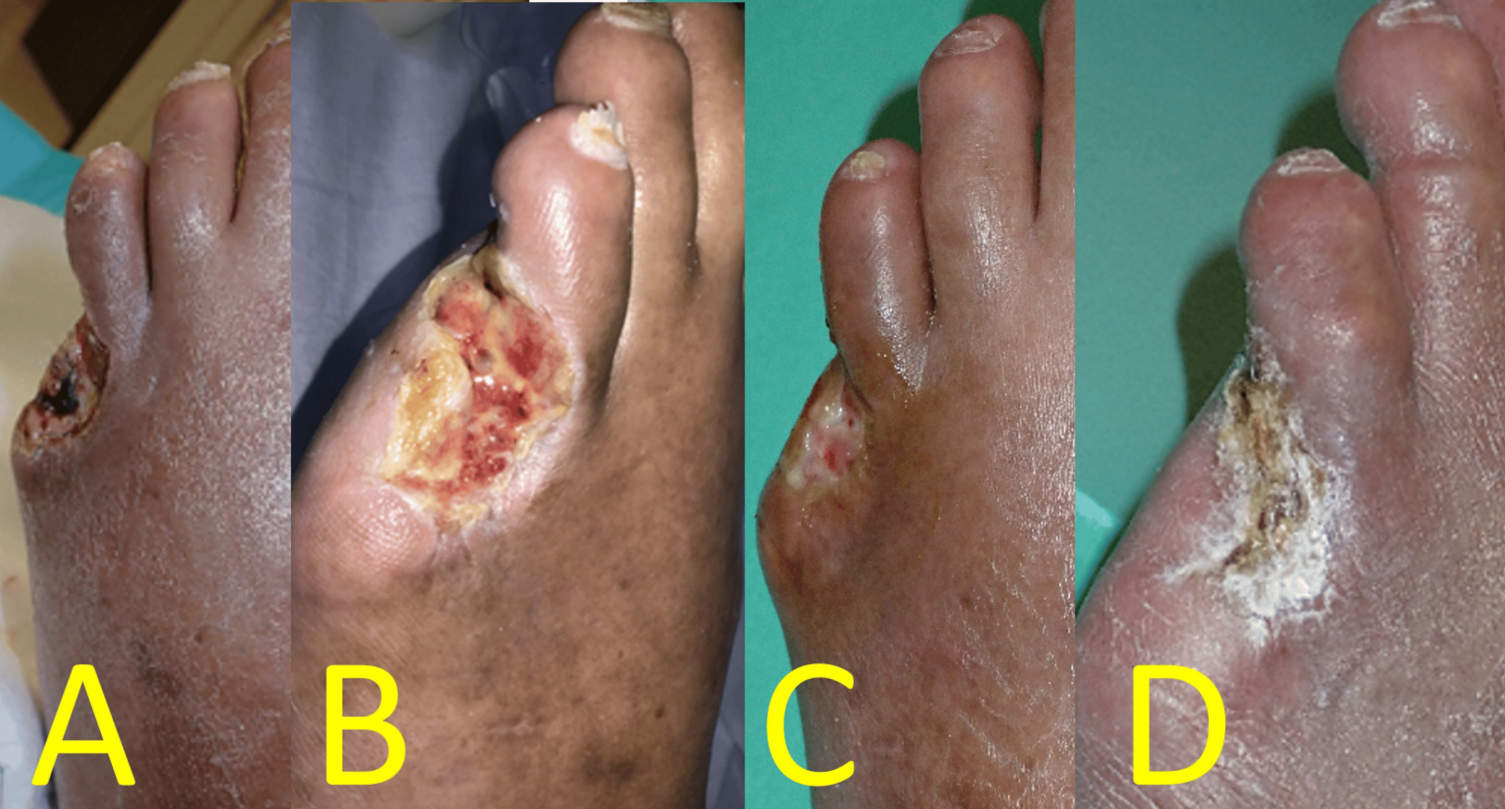
Perioperative foot findings of Case 1 (A) Wound appearance after four Rheocarna sessions. (B) Wound appearance after 11 Rheocarna sessions. (C) Wound appearance 2.5 months postoperatively. (D) Wound appearance three months postoperatively, where epithelization was completed Image credit: These images were obtained at Kagoshima City Hospital as part of routine clinical care

On postoperative day (POD) 8, skin perfusion pressures (SPPs) were 34 mmHg (dorsal) and 42 mmHg (plantar). Rheocarna therapy was initiated on POD 13 (a nonhemodialysis day following the fifth postoperative dialysis) and administered twice weekly for a total of 12 sessions. Each wound healed without severe SSI (Figures 3A-3C). The amputation wound was fully epithelialized three months after surgery (Figure 3D).

**Figure 4:**
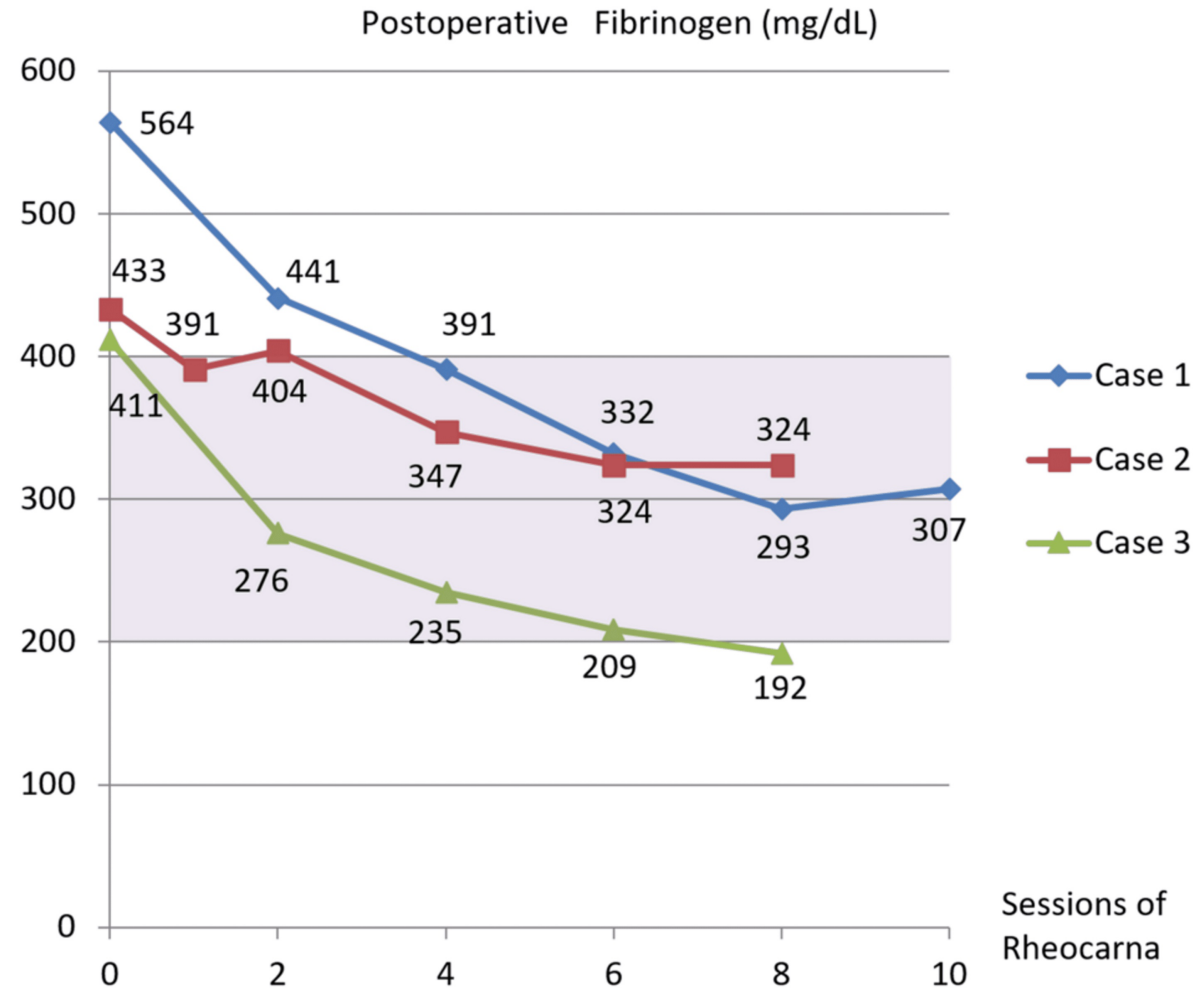
Postoperative fibrinogen changes in Cases 1-3 In all three cases, Rheocarna therapy produced an almost monotonic decrease in fibrinogen levels, maintaining them within or below the normal range (purple zone)

During the postoperative course, fibrinogen monotonously decreased to the normal range after the fourth Rheocarna (Figure 4). The patient was discharged on POD 53. However, he passed away from an acute myocardial infarction seven months postoperatively.

Case 2

**Figure 5:**
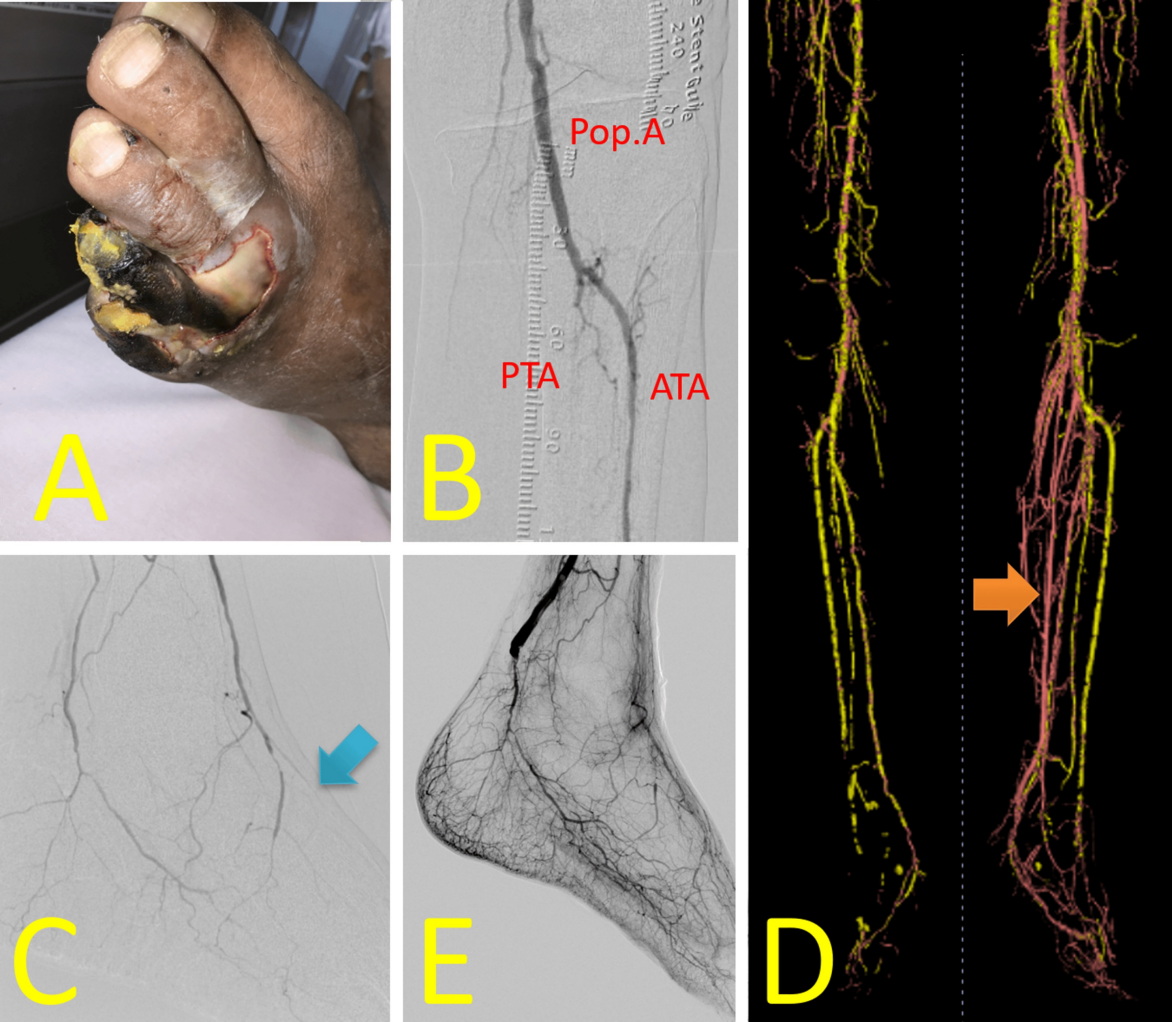
Perioperative findings of Case 2 (A) Necrosis of the left fourth and fifth toes with a red ring sign around the wound. (B) Angiography showing CTO of the left peroneal artery and PTA. (C) Angiography showing a CTO of the distal ATA that failed to be recanalized with a guidewire (blue arrow). (D) Left below-knee popliteal-to-posterior tibial artery in situ saphenous vein bypass was performed. (E) The bypass successfully increased blood flow to the PTA territory Image credit: These images were obtained at Kagoshima City Hospital as part of routine clinical care CTO: chronic total occlusion; Pop.A; popliteal artery; PTA: posterior tibial artery; ATA: anterior tibial artery

A 71-year-old Japanese man (height: 164.0 cm; weight: 45.2 kg; Table 1), who had been on hemodialysis for four years due to diabetic nephropathy, presented with necrosis of the left fourth and fifth toes (Figure 5A). He had a history of aortic valve replacement (AVR) and coronary artery bypass grafting. Ultrasound revealed occlusion of the left ATA and posterior tibial artery (PTA), and he was referred to our hospital. The purulent wound tested positive for *Escherichia coli* and Enterobacter species (W3I2fI3; stage 4). Angiography demonstrated chronic total occlusion (CTO) of the left peroneal artery (PA), PTA, and distal ATA (Figures 5B, 5C). EVT failed to revascularize the distal ATA. We then performed a left below-knee popliteal-to-posterior tibial artery in situ SV bypass (Figures 5D, 5E) and amputated the necrotic third to fifth toes, followed by weekly maintenance debridement.

**Figure 6:**
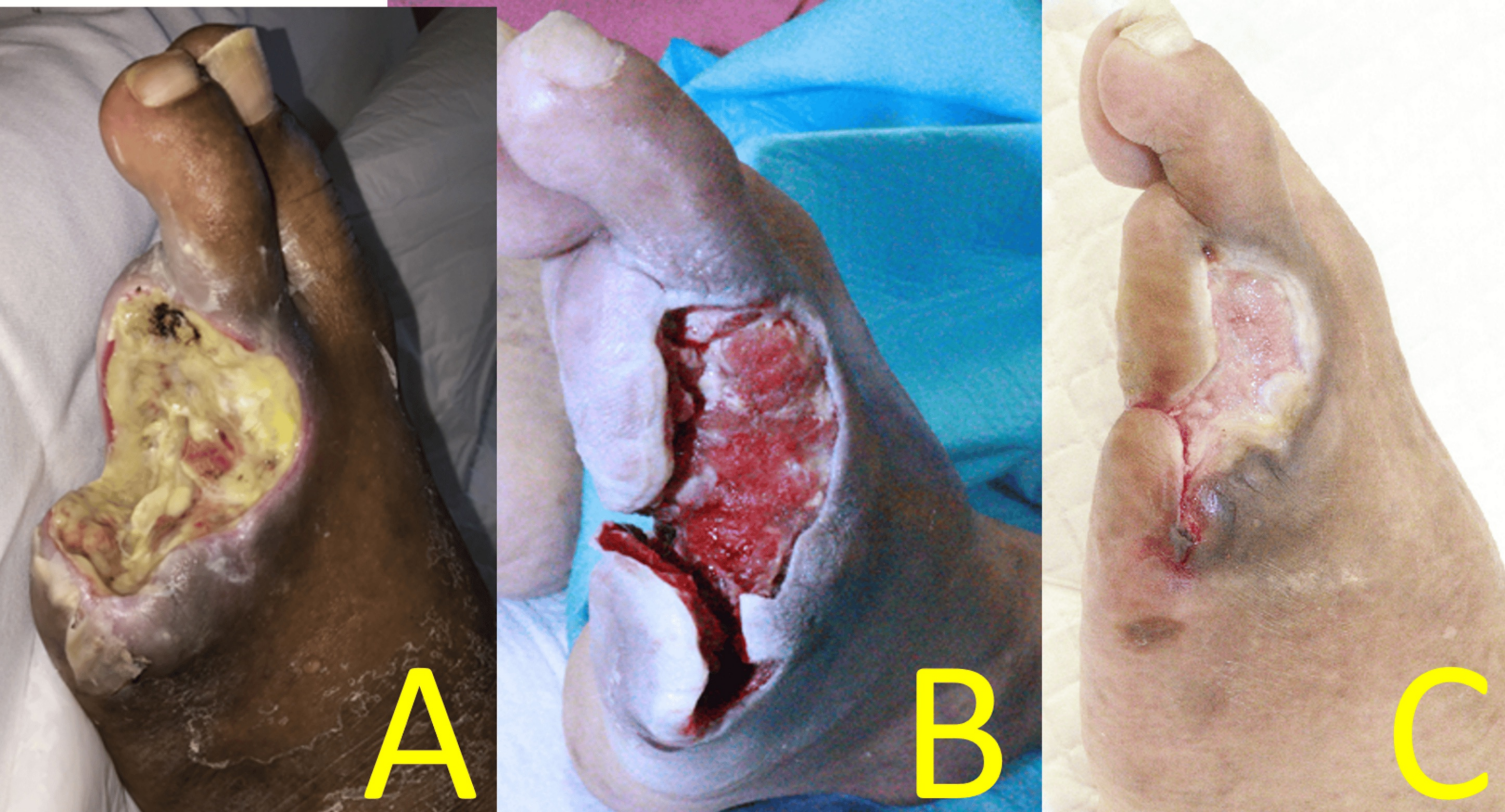
Postoperative foot findings of Case 2 (A) Wound appearance after one Rheocarna session. (B) Wound appearance after nine Rheocarna sessions. (C) Wound appearance 2.5 months postoperatively Image credit: These images were obtained at Kagoshima City Hospital as part of routine clinical care

Rheocarna therapy was initiated on POD 7 (a nonhemodialysis day following the third postoperative dialysis) and administered twice weekly for a total of eight sessions. On POD 7, left dorsal and plantar SPPs were 88 and 87 mmHg, respectively. Initially, the amputated wound was rich in infected granulation tissue (Figure 6A). However, after wound infection was controlled (Figure 6B), negative pressure wound therapy was initiated on POD 29, and the wound contracted. Each wound healed without SSI. The patient was transferred to the referring hospital on POD 35. The amputation wound was epithelialized 2.5 months after surgery (Figure 6C).

**Figure 7:**
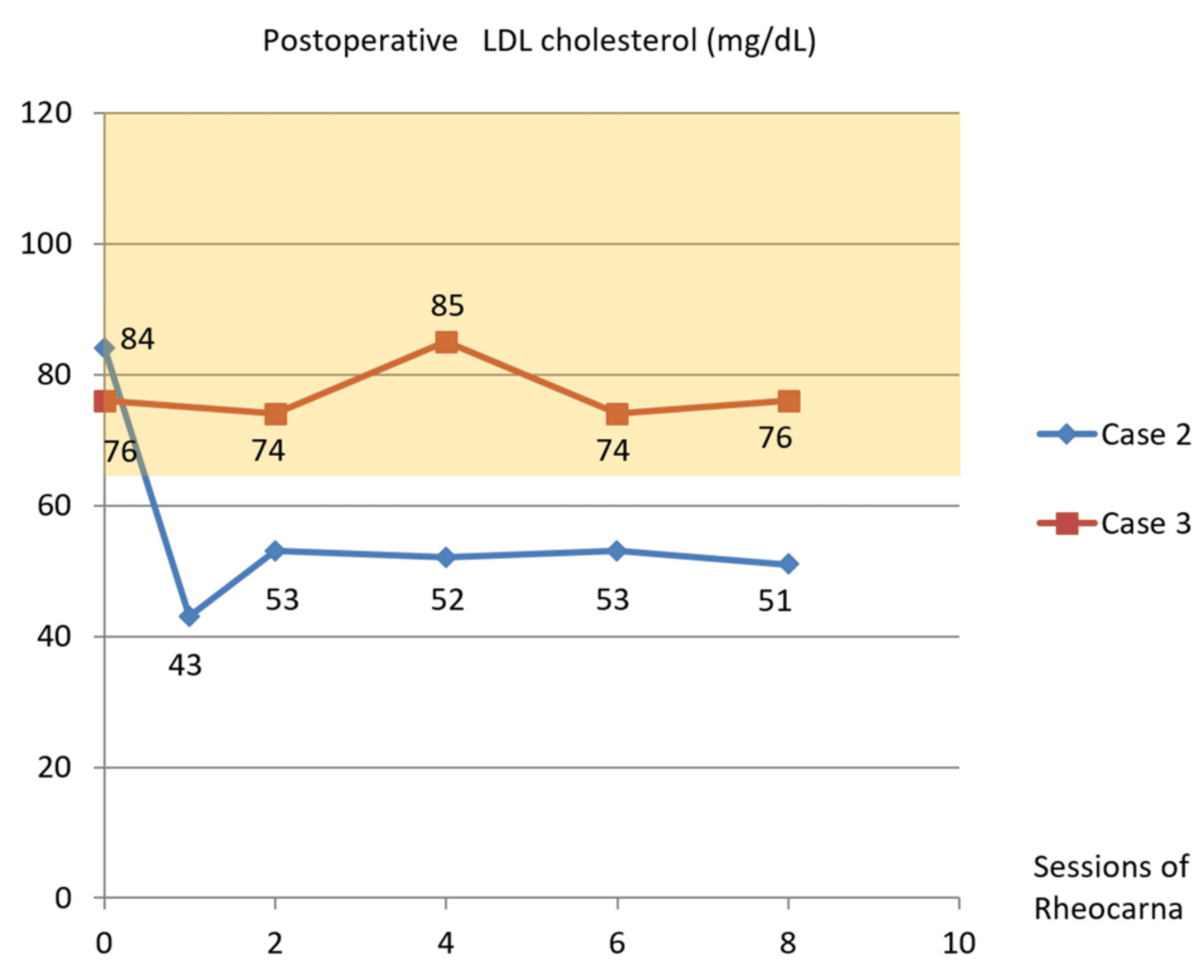
Postoperative LDL cholesterol changes in Cases 2 and 3 In Case 2, LDL cholesterol levels remained below the normal range (dark yellow zone), and in Case 3, they remained lower normal range LDL: low-density lipoprotein

**Figure 8:**
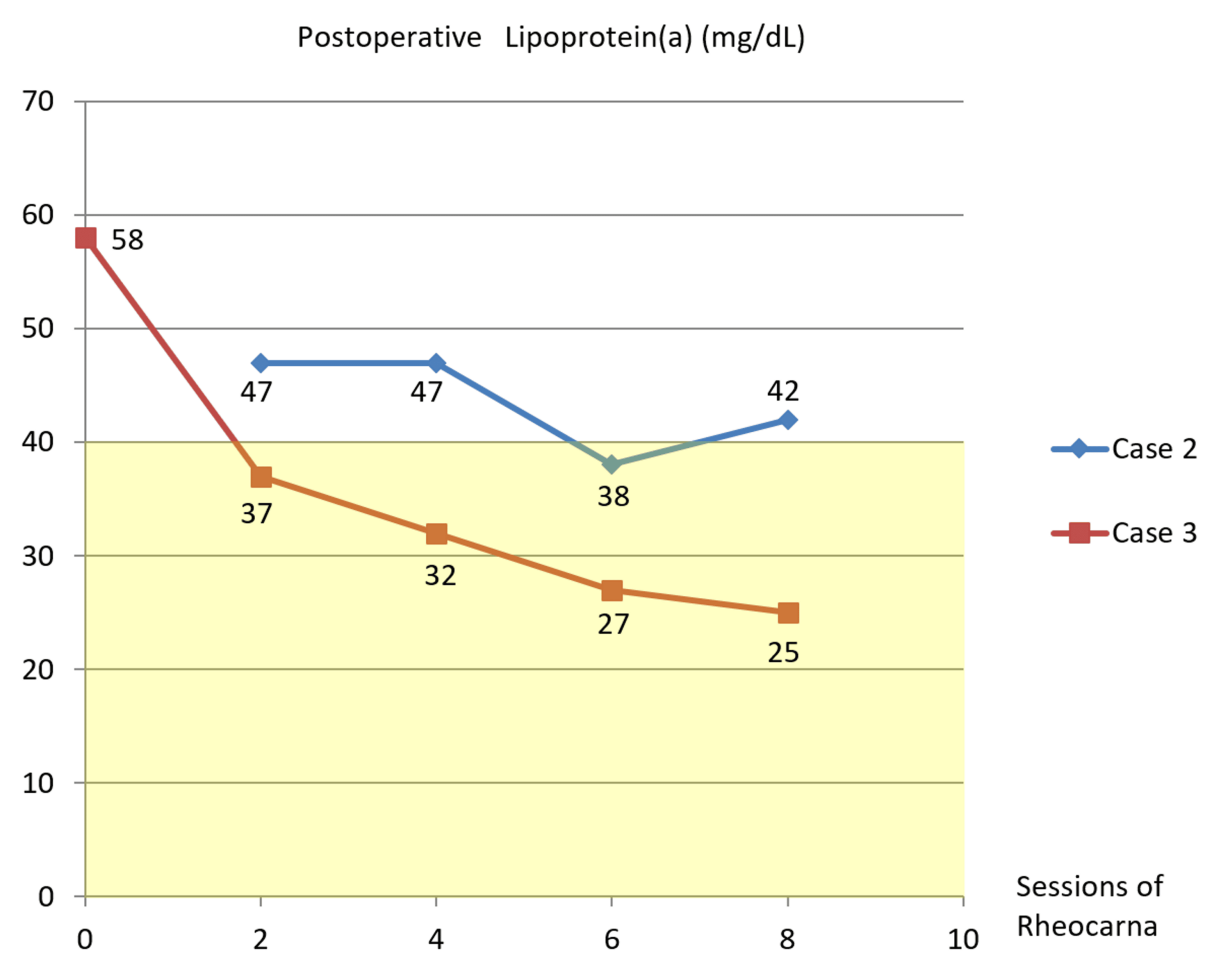
Postoperative lipoprotein(a) changes in Cases 2 and 3 In Case 2, lipoprotein(a) decreased to around the upper limit of the normal range (yellow zone), while in Case 3, it monotonically decreased to within the normal range

During the postoperative course, fibrinogen decreased to the normal range after the first Rheocarna therapy (Figure 4). LDL-cholesterol remained lower normal range (Figure 7), and lipoprotein(a) monotonically decreased to within the normal range (Figure 8). He passed away due to food aspiration five months postoperatively.

Case 3

**Figure 9:**
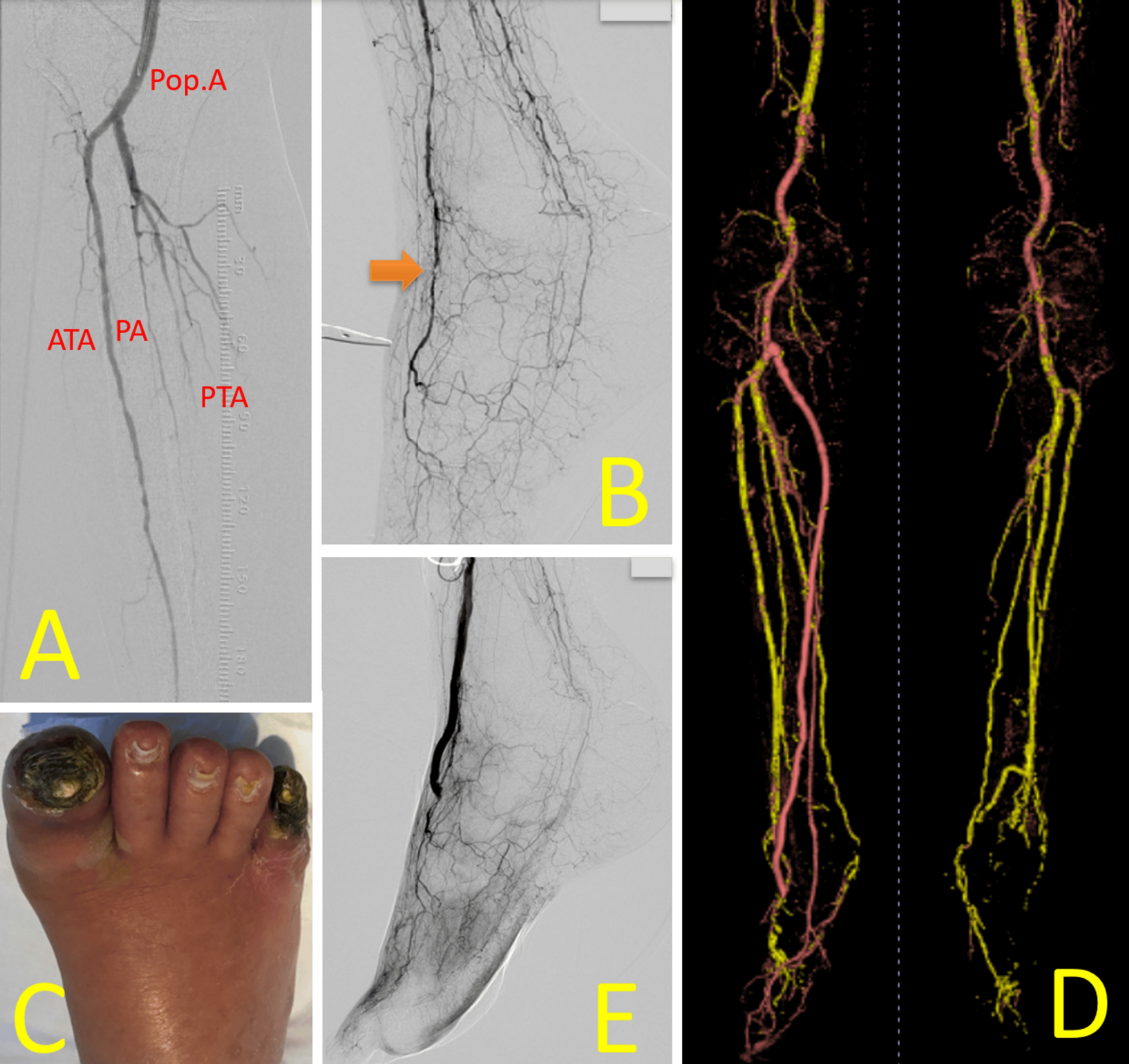
Perioperative findings of Case 3 (A) EVT achieved revascularization of the PA from 99% to 25%. (B) EVT failed to recanalize the chronic total occlusion of the distal ATA (orange arrow) or PTA. (C) Necrosis of the first and fifth toes progressed, complicated by larger cellulitis. (D) Right below-knee popliteal-to-dorsalis pedis artery in situ saphenous vein bypass was performed. (E) The bypass successfully increased blood flow in the ATA territory Image credit: These images were obtained at Kagoshima City Hospital as part of routine clinical care EVT: Endovascular therapy; Pop. A: popliteal artery; ATA: anterior tibial artery; PTA: posterior tibial artery; PA: peroneal artery

A 79-year-old Japanese man (height: 168.0 cm; weight: 50.0 kg; Table 1), who had been on hemodialysis for eight years due to chronic kidney disease, presented with distal phalanx exposure of the right first toe and was referred to our hospital. He had a history of AVR, tricuspid valve annuloplasty, and interstitial pneumonia. The right first and fifth toes were necrotic. EVT achieved revascularization of the PA from 99% to 25% (Figure 9A), but failed to recanalize the CTO in the distal ATA and PTA (Figure 9B). Necrosis worsened, and larger cellulitis developed (Figure 9C). The purulent wound tested positive for *Staphylococcus aureus* and *Streptococcus oralis *(W2I3fI3; stage 4). We then performed a right below-knee popliteal-to-dorsal artery in situ SV bypass (Figure 9D), successfully increasing blood flow in the ATA territory (Figure 9E). The necrotic first and fifth toes were amputated, and weekly maintenance debridement was carried out.

**Figure 10:**
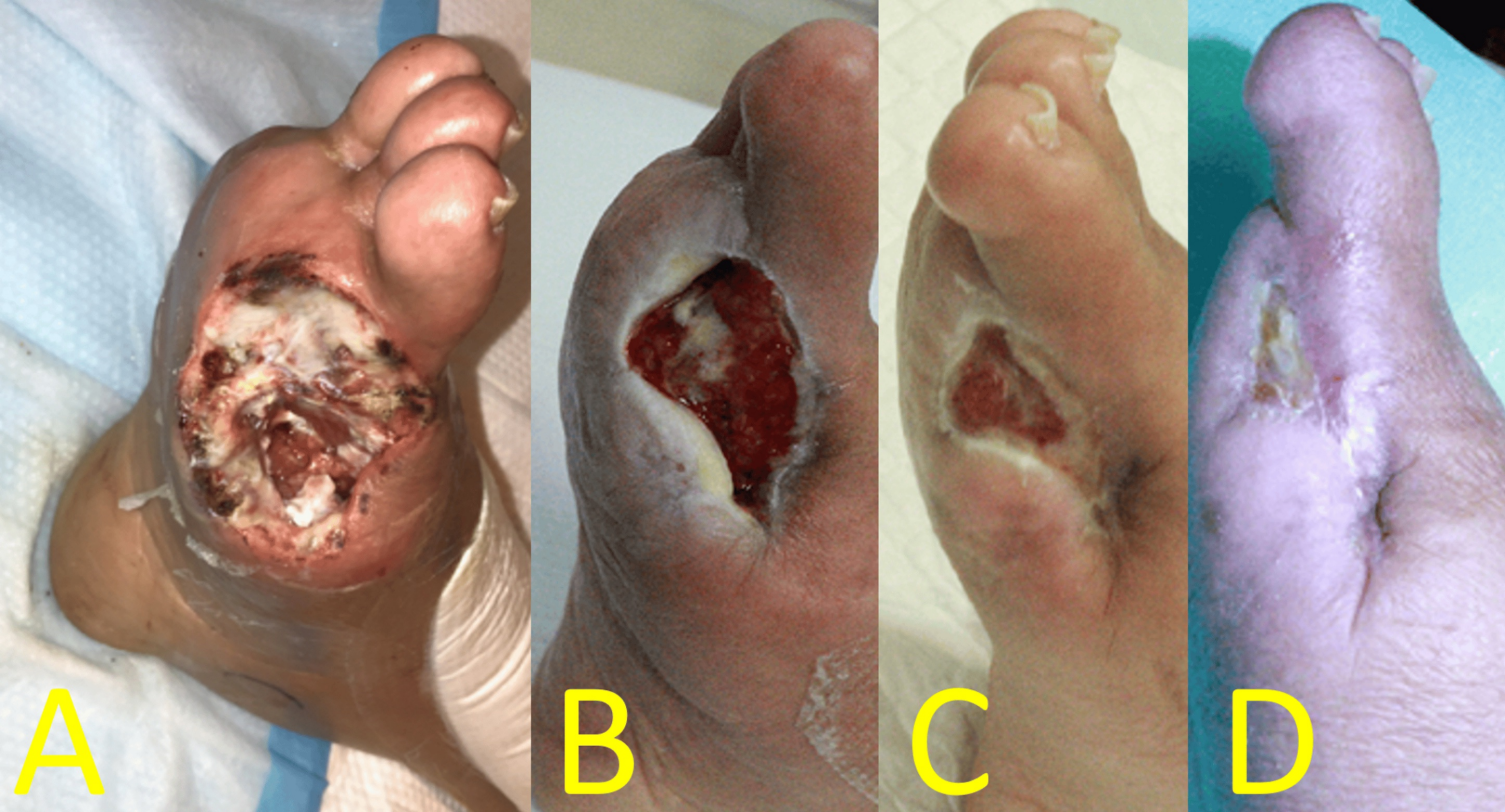
Postoperative foot findings of Case 3 (A) Wound appearance before initiation of Rheocarna. (B) Wound appearance after eight Rheocarna sessions, where cellulitis resolved. (C) Wound appearance two months postoperatively. (D) Wound appearance three months postoperatively, where epithelization was completed Image credit: These images were obtained at Kagoshima City Hospital as part of routine clinical care

On POD 7, right dorsal and plantar SPPs improved to 58 and 47 mmHg, respectively. Rheocarna therapy was initiated on POD 9 (a nonhemodialysis day following the fourth postoperative dialysis) and administered twice weekly for a total of nine sessions. The amputation wound was initially rich in infected granulation tissue (Figure 10A), but each wound healed without SSI (Figures 10B, 10C). The patient was discharged on POD 42. The amputation wound was completely epithelialized three months after surgery (Figure 10D).

During the postoperative course, fibrinogen decreased to the normal range after the second Rheocarna therapy (Figure 4). LDL cholesterol was kept under normal range (Figure 7), and lipoprotein(a) was kept within normal range (Figure 8). He remained free from SSI or graft occlusion for one year and two months postoperatively.

## Discussion

WIfI clinical stage 4 CLTI represents the highest risk category for major foot amputation and is associated with significantly reduced quality of life. In addition, bacterial infection in the affected foot can be life-threatening. The Surgical Reconstruction Versus Peripheral Intervention in Patients With Critical Limb Ischemia study [7] in Japan demonstrated that surgical revascularization is more favorable than EVT in patients with: 1) WIfI W3 classification, 2) fI-2/3, 3) a history of ipsilateral minor amputation, 4) a history of revascularization after CLTI onset, and 5) bilateral CLTI. Based on these criteria, we performed urgent distal bypass using autologous vein grafts in all three patients. Additionally, immediate minor amputations were performed by plastic and reconstructive surgeons. Purulent discharge and infected granulation tissue (Figures 3A, 6A, 10A) accumulated at the wound edges postdebridement, delaying wound healing. Therefore, weekly maintenance debridement was necessary in conjunction with daily foot care.

Bypass graft failure, SSI, and delayed wound healing are critical concerns in distal bypass surgery for patients on hemodialysis. They have poor distal runoff due to a more affected pedal arch [8], marked arterial calcification, and a hypercoagulable state [9]. These conditions may lead to anastomotic disruption and early graft occlusion. In Cases 2 and 3, the patients were notably thin (Table 1), and therefore, the skin incisions were placed away from the dorsal artery and PTA to prevent graft exposure in the event of SSI. Careful selection and duration of antibiotic therapy, along with meticulous anastomotic wound management, are also essential. All three CLTI cases involved toe osteomyelitis and cellulitis due to several types of pathogens. Therefore, we administered broad-spectrum antibiotics, including Vancomycin hydrochloride, Meropenem hydrate, Tazobactam/Piperacillin hydrate, or Cefepime dihydrochloride hydrate, depending on the identified pathogens, continuing treatment until epithelialization was nearly complete and serum C-reactive protein levels decreased to <1.0 mg/dL.

In addition to surgical technique and antibiotics, LDL apheresis using Rheocarna may also play an important role in avoiding SSI and delayed wound healing. Rheocarna is composed of a polypropylene column filled with cellulose beads averaging 450 μm in diameter. The bead surfaces are biochemically modified with negatively charged dextran sulfate and hydrophobic L-tryptophan. As blood flows through the column, LDL cholesterol containing positively charged apolipoprotein B binds to the beads, while fibrinogen is captured through hydrophobic interactions with L-tryptophan [10]. Thus, Rheocarna therapy improves oxidative stress [11-13], blood viscosity and fluidity [14], and endothelial function [13]; enhances anti-inflammatory effects [11,14]; and stimulates production of vasodilators [15,16], such as bradykinin and endogenous nitric oxide. Collectively, these mechanisms improve microcirculation by reducing local wound inflammation [17], promoting faster wound healing [10], and helping prevent anastomotic disruption and early graft occlusion.

Since 2021, Rheocarna therapy has been reimbursed under Japan’s national health insurance for patients with LEAD who are 1) Fontaine stage IV or 2) unresponsive to surgery and EVT due to infrapopliteal or extensive arterial occlusion, and resistance to conventional medical therapy [5]. According to the 2024 guideline, spontaneous wound healing is generally expected at an SPP threshold of 40 mmHg [18]. In our study, Rheocarna therapy was introduced in Case 1 because neither postoperative dorsal nor plantar SPP exceeded 40 mmHg. However, in patients with uncontrolled diabetes, hyperfibrinogenemia due to deep-seated infection, or those undergoing hemodialysis, improved SPPs after distal bypass procedures do not always result in marked wound healing. Therefore, Rheocarna therapy was also applied to Cases 2 and 3. In particular, because hemodialysis patients already possess vascular access suitable for apheresis, introducing Rheocarna therapy after distal bypass is reasonable.

**Figure 11:**
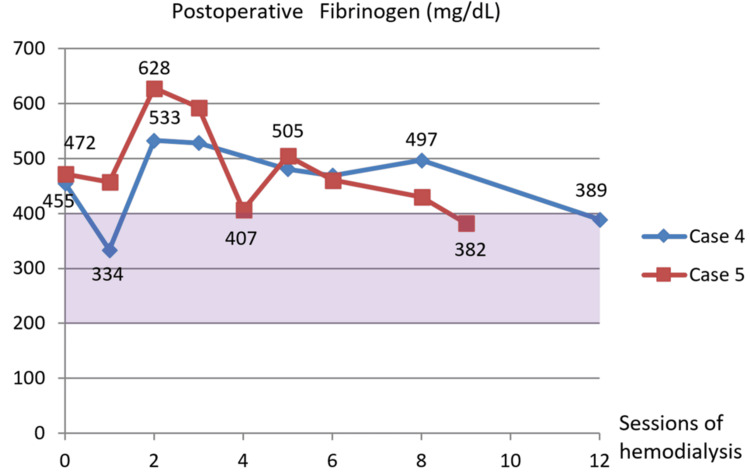
Postoperative fibrinogen changes in Cases 4–5. Without Rheocarna, postoperative fibrinogen levels remained above the normal range (purple zone) until the eighth hemodialysis session.

In our three patients with WIfI clinical stage 4 CLTI (Cases 1-3), Rheocarna therapy appeared to reduce fibrinogen levels to within or below the normal range (Figure 4). We compared these patients with two other patients with WIfI clinical stage 3 CLTI (Cases 4 and 5; Table 2) who were on hemodialysis and did not receive Rheocarna therapy at our hospital. In Cases 4 and 5, fibrinogen levels lingered above the normal range and in the upper normal range until the ninth postoperative hemodialysis session at least (Figure 11). Moreover, in Case 2, Rheocarna therapy may have reduced LDL cholesterol to below the normal range (Figure 7), and in Cases 2 and 3, lipoprotein(a) was also decreased to within the normal range (Figure 8). In Case 1, postoperative LDL cholesterol and lipoprotein(a) were not measured. Lipoprotein(a) is a modified form of LDL cholesterol, and both LDL cholesterol and lipoprotein(a) contain one molecule of apolipoprotein B. Therefore, if their apolipoprotein is positively charged, they can be adsorbed by Rheocarna. Based on our initial three successful cases, Rheocarna therapy may be a promising adjunctive therapy for microcirculation improvement and may warrant broader application following distal bypass procedures for future patients.

**Table 2:** Preoperative characteristics of other two hemodialysis patients (Cases 4 and 5) with WIfI clinical stage3 CLTI, who did not receive Rheocarna therapy BMI: body mass index; HD: hemodialysis; WBC: white blood cell; CRP: C-reactive protein; Alb: albumin; HbA1c: hemoglobin A1c; NGSP: National Glycohemoglobin Standardization Program; LDL-C: low-density lipoprotein cholesterol; ABI: ankle brachial index; SPP: skin perfusion pressure; DP: dorsal pedis; PP: plantar pedis; WIfI: Wound, Ischemia and foot Infection

Case	Age/sex	BMI (kg/m^2^)	Affected foot	HD period (years)	WBC (/μL)	CRP (mg/dL)	Alb (g/dL)	HbA1c (NGSP) (%)	Glycoalbumin (%)	LDL-C (mg/dL)	Fibrinogen (mg/dL)	ABI of the affected foot	SPP of the affected foot (mmHg)		WIfI classification
													DP	PP	
4	84/Male	19.3	Left	1	4,100	0.78	3.5	6	Not measured	76	455	Unmeasurable	15	20	W1I3fI1
5	76/Male	22	Right	7	5,300	2.41	3.4	5.5		158	496	0.8	48	20	W2I1fI1

On the other hand, Rheocarna should be used with caution in patients at risk of hypotension. Angiotensin-converting enzyme inhibitors (ACE-Is) [5] and unresolved deep infections [19] can increase circulating bradykinin and cause hypotension during apheresis. None of the three patients was taking ACE-Is. The foot infections in Cases 2 and 3 were classified as Grade 3, the most severe grade, but minor amputations, daily wound care, and broad-spectrum antibiotics gradually stabilized their vital signs and reduced white blood cell count and C-reactive protein levels. This is because Rheocarna was initiated as early as POD 7 in Case 2, and no significant hemodynamic changes occurred during apheresis.

Limitations

This is a retrospective study with a small sample size (n = 3) and lacks a long-term control group to compare wound recurrence rates.

## Conclusions

We treated WIfI clinical stage 4 CLTI using popliteal-to-distal bypass, minor amputation, debridement, and Rheocarna therapy in three hemodialysis patients. Rheocarna therapy may reduce postoperative fibrinogen, LDL cholesterol, and lipoprotein(a), improving microcirculation and avoiding bypass graft failure, SSI and delayed wound healing. Given the limited reports on the use of Rheocarna after distal bypass, it is important to accumulate more cases and long-term outcome data. Further multicenter studies or registry data are needed to validate the optimal timing and frequency of Rheocarna therapy.
